# The self-directed patient in guided internet-delivered CBT: a qualitative study of patient role expectations

**DOI:** 10.3389/fpsyg.2026.1924186

**Published:** 2026-08-19

**Authors:** Beate Standal, Robin Maria Francisca Kenter, Monika Knudsen Gullslett, Tine Nordgreen, Jørn Heggelund, Inger Lise Teig

**Affiliations:** 1 Department of Global Public Health and Primary Care, Faculty of Medicine, University of Bergen, Bergen, Norway; 2 Research Centre for Digital Mental Health Services, Division of Psychiatry, Haukeland University Hospital, Bergen, Norway; 3 Norwegian Centre for E-health Research, University Hospital of Northern Norway, Tromsø, Norway; 4 Department of Mental Health, Faculty of Medicine and Health Sciences, Norwegian University of Science and Technology, Trondheim, Norway

**Keywords:** autonomy, digital mental health, guided iCBT, motivation, patient role, qualitative research, self-determination theory

## Abstract

**Background:**

Therapist-guided internet-delivered cognitive behavioral therapy (guided iCBT) is an effective and scalable treatment for anxiety and depression. Central to this format is the expectation that patients work independently with therapeutic content. However, limited attention has been paid to how healthcare professionals and patients within guided iCBT understand the patient role.

**Objective:**

This study explores how healthcare professionals and patients understand the patient role in guided iCBT, using self-determination theory to examine how autonomy, competence, and relatedness shape experiences of the patient role.

**Methods:**

A qualitative study was conducted in Norwegian specialist mental health services, where guided iCBT is delivered as part of routine care, combining interviews and observations with healthcare professionals (*n* = 31) and free-text responses from patients (*n* = 296). Healthcare professional data were analyzed using reflexive thematic analysis and patient responses using inductive qualitative content analysis.

**Results:**

Healthcare professionals constructed an ideal of the “motivated and self-driven” patient, reflecting an implicit norm of self-direction as a core expectation of the patient role. Patients varied in their ability to enact this role: some described themselves in ways that aligned with this expectation, emphasizing independence and skill use, while others experienced tensions between demands for autonomy, their perceived competence, and their need for structure and relational support. Although many patients reported gaining skills, the treatment was also described as demanding and, at times, overwhelming, with difficulties in participation frequently framed as personal failure, accompanied by self-criticism and guilt.

**Conclusion:**

Guided iCBT relies on an implicit expectation that patients will be self-directed, but patients’ experiences vary depending on how well they can assume this role. From a self-determination theory perspective, autonomy may function not only as a supportive resource but also as a demand when support for competence and relatedness is insufficient. These findings highlight the importance of carefully structuring and supporting expectations of self-direction within guided iCBT.

## Introduction

Internet-delivered psychological treatments have been extensively studied over the past decades and are now well established as effective interventions for common mental health problems (Vernmark et al., 2024; Andersson et al., 2019). Therapist-guided internet-delivered cognitive behavioral therapy (guided iCBT), in which patients complete structured online modules with asynchronous written therapist support, has been shown to produce outcomes comparable to face-to-face therapy for anxiety and depressive disorders (Andersson et al., 2019; Moshe et al., 2021; Biagianti et al., 2023; Hedman-Lagerlöf et al., 2023; Rosenström et al., 2025). As guided iCBT becomes increasingly integrated into routine mental health care, its scalability and potential to improve access suggest continued expansion (Duffy et al., 2025; Thew, 2020; Vernmark et al., 2024). At the same time, the shift from face-to-face to digitally delivered therapy may alter expectations of what it means to be a patient, particularly regarding autonomy, responsibility, and engagement.

As guided iCBT becomes more widely used, patient engagement becomes central to how this form of treatment functions. While active participation and the application of therapeutic techniques in daily life are central to many forms of psychotherapy, guided iCBT places greater emphasis on patients’ independent completion of structured modules and sustained engagement over time. This suggests that guided iCBT entails specific expectations about patients’ roles and responsibilities during treatment.

Studies describing patients in guided iCBT suggest that patients seeking internet-based treatment are broadly comparable to those in face-to-face therapy. However, one earlier study suggests they may be older, more often female, and more highly educated (Titov et al., 2010a). More recent evidence indicates largely similar baseline characteristics overall, with some indications of longer symptom duration and lower prior treatment use in digital treatment samples (Aemissegger et al., 2022). Findings across recruitment pathways are mixed, with some studies suggesting higher symptom severity among actively help-seeking individuals and others showing no differences across referral routes (Lindner et al., 2015; Bjarke et al., 2025). Together, findings suggest that patients entering guided iCBT represent a heterogeneous group, which may influence how expectations regarding participation and responsibility are experienced.

Research has also investigated patients’ experiences with guided iCBT. Patients are generally satisfied, reporting perceived support from practitioners and valuing aspects of the online format such as flexibility and accessibility (Perera-Delcourt and Sharkey, 2019; Thew, 2020). However, negative experiences are also documented, including stress, frustration, and perceived pressure related to treatment tasks. In some cases, these extend beyond subjective experiences to include symptom deterioration or the emergence of new symptoms in a subset of patients (Rozental et al., 2015; Ebert et al., 2016; Fenski et al., 2025). Overall, these findings indicate that patients’ experiences of guided iCBT are heterogeneous, with many benefiting from the autonomy afforded by the format, while others may find aspects of the intervention demanding and prefer more synchronous contact or additional support (Etzelmueller et al., 2018; Wiberg et al., 2025).

Concerns about engagement further reflect this tension between autonomy and treatment demands. Dropout rates in iCBT are often reported to be higher than in face-to-face therapy, particularly in low-support formats; however, findings remain inconsistent and appear to depend on intervention characteristics such as level of guidance (Tong et al., 2026; Linardon et al., 2025). Efforts to enhance motivation have shown mixed results, with some benefit in self-guided formats but limited additional effect in guided iCBT (Titov et al., 2010b; Horse et al., 2023; Peynenburg et al., 2022; Soucy et al., 2021). At the same time, features inherent to iCBT, such as structured modules, asynchronous communication, and varied referral pathways, may shape both engagement and the experience of motivation. For instance, self-referred patients often show better outcomes, potentially reflecting higher intrinsic motivation, whereas non-responders more frequently report difficulty maintaining engagement and a preference for more synchronous support (Bjarke et al., 2025; Wiberg et al., 2025; Kok et al., 2017).

While prior research in guided iCBT has largely focused on treatment outcomes, patient characteristics, and patient behavior, less attention has been paid to how expectations regarding patients’ roles are understood, experienced, and negotiated within the treatment context.

The concept of the patient role offers a useful lens for examining these issues. It can be traced to Parsons’ (1951) notion of the “sick role,” which frames illness as a socially defined role with specific rights and responsibilities that shape expected behaviors when ill. In this framework, patients are seen as relatively passive recipients of care, expected to comply with medical advice and authority.

However, this view has evolved, with greater emphasis on patient autonomy, responsibility, and active participation in managing health (Burnham, 2012). This shift is particularly relevant in digital health contexts, where patients are increasingly expected to manage their care actively, reflecting a broader trend toward the “digitally engaged” and responsible patient (Lupton, 2013; Petrakaki et al., 2018).

Guided iCBT exemplifies this shift by facilitating autonomy and flexibility while also requiring sustained motivation, self-regulation, and active participation. In this study, the patient role is understood as a socially constructed set of expectations. This understanding draws on social constructionist perspectives, which view social roles and social reality as produced and reproduced through ongoing processes of interpretation and interaction (Berger and Luckmann, 1966). From this perspective, roles are shaped through the interaction between institutional expectations and individuals’ own interpretations of and responses to those expectations.

Following Wenger (1999), negotiation is understood broadly as the processes through which expectations, responsibilities, meanings, and ways of acting are interpreted, adjusted, accommodated, contested, and aligned in practice. Negotiation does not refer only to explicit discussions or formal decision-making, but also to the everyday ways individuals respond to, reproduce, struggle with, or adapt to expectations. In the present study, this concept is used analytically to understand how participants described, interpreted, and responded to expectations associated with treatment participation. Accordingly, patient roles are understood as negotiated through the expectations associated with treatment participation and the ways these expectations are interpreted and enacted by healthcare professionals and patients. Understanding these processes requires moving beyond descriptions of adherence and outcomes to examine how expectations regarding participation, responsibility, autonomy, and support are constructed, interpreted, and experienced within guided iCBT.

### Self-determination theory as a framework for understanding the patient role in guided iCBT

Self-Determination Theory (SDT) (Deci and Ryan, 1985; Ryan and Deci, 2017) provides a useful framework for understanding motivation and engagement in guided iCBT, particularly given its emphasis on autonomy and active participation. SDT distinguishes between *autonomous motivation*, driven by personal values and intrinsic interest, and *controlled motivation*, driven by external pressures or demands. Autonomous motivation is generally associated with more favorable psychological outcomes and enhanced well-being (Deci and Ryan, 2008; Ntoumanis et al., 2021; Slemp et al., 2024), suggesting that the way motivation is supported in guided iCBT may be critical for both engagement and the treatment experience.

According to SDT, motivation depends on the extent to which the three basic psychological needs autonomy, competence, and relatedness are supported. Autonomy refers to experiencing a sense of ownership over one’s actions. It is fostered when individuals perceive activities as meaningful and personally interesting and undermined when individuals feel pressured or controlled. In guided iCBT, the flexible and self-paced structure may support autonomy by allowing individuals to engage with treatment in ways that align with their preferences and daily routines. Competence reflects a sense of effectiveness and capability, supported through managing challenging tasks and experiencing improvement. In guided iCBT, competence may be supported through structured modules that provide psychoeducation and exercises aimed at skill acquisition and reinforcing a sense of progress over time. Relatedness involves feelings of being connected to, understood by, and cared for by others, and is supported through experiences of warmth and acceptance. In the context of guided iCBT, relatedness may be fostered through interactions with a therapist, even when these are brief or mediated digitally, by providing support, encouragement, and validation (Ryan and Deci, 2017). Some research also suggests that patients may develop a sense of connection to the treatment program itself, indicating that experiences of relatedness in guided iCBT may not be limited to the therapist relationship alone (Zalaznik et al., 2025).

Importantly, SDT conceptualizes motivation as shaped by how social environments support or hinder individuals’ basic psychological needs. In applied contexts, this includes how organizational structures, expectations, and roles may influence opportunities to satisfy needs. However, individuals may vary in how they experience and respond to such contextual conditions, which in turn affects their sense of autonomy, competence, and relatedness. From this perspective, the patient role can be understood as a socially and contextually shaped set of expectations that influences how these needs are supported or undermined. As a result, autonomy may be experienced as either empowering or pressuring, competence as supported or diminished, and relatedness as either sufficient or lacking.

Despite increasing interest in motivation and relational factors in digital therapy, relatively little research has examined how patients and healthcare professionals understand and negotiate the patient role in guided iCBT. Much of the existing literature focuses on treatment outcomes, adherence, and user experiences, with less attention to how expectations regarding roles and responsibilities are constructed and interpreted within the treatment context (Andersson et al., 2019; Bendelin et al., 2011). This gap is important, as how the patient role and its associated expectations are understood and negotiated may shape how individuals experience autonomy, seek support, and manage treatment demands. Applying an SDT framework enables a more in-depth exploration of these processes by linking participants’ understandings of expectations and responsibilities to underlying motivational dynamics.

The present study addresses this gap by qualitatively examining how the patient role is understood and negotiated in guided iCBT, with particular attention to how expectations of autonomy, responsibility, and support are articulated by healthcare professionals and experienced by patients. Guided by an SDT perspective, the study explores how these expectations relate to motivational processes within the treatment context. Specifically, the study addresses the following questions:

1. How do patients and healthcare professionals understand and respond to expectations associated with the patient role in guided iCBT?
2. How are expectations regarding autonomy, responsibility, and support articulated, interpreted, and responded to?
3. How can these expectations be understood in relation to motivational processes described in self-determination theory (SDT)?

## Materials and methods

### Study design

This study drew on two qualitative datasets collected within the implementation of guided iCBT in Norwegian specialist mental health services: (1) interviews and observations involving healthcare professionals (*n* = 31), and (2) free-text responses from a post-treatment patient survey (*n* = 296 patients providing analyzable responses).

The analyses were conducted sequentially. First, interviews and observations with healthcare professionals were analyzed inductively to explore experiences with and understandings of guided iCBT in practice. Themes relating to the patient’s role informed a subsequent analysis of patient free-text responses, enabling a more focused exploration of patients’ descriptions of treatment experiences, efforts, and challenges.

The final themes were interpreted using self-determination theory (SDT).

### Relationship between the datasets

The two datasets differed substantially in purpose and depth and were therefore treated as complementary rather than equivalent sources of evidence. Interviews and observations with healthcare professionals provided detailed accounts of how expectations regarding patient motivation, responsibility, and suitability were constructed within guided iCBT services. In contrast, patient survey comments provided brief retrospective accounts of treatment experiences. Integration occurred during interpretation, where healthcare professionals’ descriptions of patient-role expectations were examined alongside patients’ accounts of treatment participation, challenges, and support needs. The healthcare professional and patient datasets were drawn from largely overlapping service contexts, with three of the four clinics contributing patient survey data also represented in the healthcare professional dataset. Individual patients and healthcare professionals could not be linked and were not sampled as matched participants.

### Study context

This study examined the patient role in the implementation and scale-up of guided iCBT in Norwegian specialist health services. Introduced in 2013, guided iCBT has gradually expanded from pilot use to routine care and now includes diagnosis-specific programs for depression, social anxiety disorder, and panic disorder. Within this context, the study explored how patients and healthcare professionals understood, interpreted, and experienced the patient role, how expectations regarding autonomy, responsibility, and support were articulated, and how these expectations could be interpreted in relation to motivational processes described in SDT. The effectiveness of guided iCBT in Norwegian routine care is supported by a growing body of evidence (Bjarke et al., 2025; Khan et al., 2025; Nordgreen et al., 2018a, 2018b; Nordgreen et al., 2019).

### Guided iCBT intervention

Treatment began with digital assessments followed by a face-to-face assessment interview with a therapist. Eligible patients met the diagnostic criteria for depression, social anxiety disorder, or panic disorder; had access to secure electronic identification; and possessed sufficient proficiency in written Norwegian and digital literacy to use the platform and complete the treatment activities.

Patients were granted access to a diagnosis-specific treatment program delivered through a secure digital platform. The depression program consisted of eight modules, whereas the panic disorder and social anxiety programs consisted of nine modules. Patients were expected to spend 7–10 days on each module, with treatment lasting up to 14 weeks. Progression to subsequent modules was determined by the therapist based on completion of exercises and treatment progress, and patients received automated SMS notifications when a new module became available or when they had received a message within the program.

The programs were based on cognitive behavioral therapy (CBT) principles and included psychoeducation, written exercises, homework assignments, and symptom-monitoring. Exercises included behavioral activation, situational exposure, and interoceptive exposure as part of patients’ independent work within the modules. Modules contained substantial written content. The treatment programs were standardized and the core treatment content could not be modified, although therapists could provide individualized guidance, encouragement, and suggestions through written communication.

Therapist support was provided primarily through asynchronous written communication within the platform. Patients could send messages at any time, and therapists typically responded within three working days. If a patient was inactive for approximately 1 week, therapists usually initiated contact via the platform. Weekly symptom monitoring was conducted using standardized questionnaires, with predefined procedures followed if responses indicated elevated suicidal ideation. Additional support, including telephone or face-to-face consultations, could be provided when clinically indicated. A later study from one of the included iCBT clinics, using a newer treatment platform, found that messaging and monitoring activities required approximately 15 min of therapist time per patient per week (Brunner et al., 2026).

Further details on the intervention, implementation procedures, and outcome measures are described elsewhere (Khan et al., 2025; Nordgreen et al., 2018a, 2018b; Nordgreen et al., 2019).

### Data collection

#### Interviews with healthcare professionals

Semi-structured interviews were conducted to explore healthcare professionals’ experiences with implementing guided iCBT. The interview guide was developed collaboratively by the research team. Participants were asked about their experiences with guided iCBT, their views on delivering it, organizational requirements for its use, conditions under which it might be considered equivalent to other treatment modalities, and strategies for increasing its uptake. The full interview guide is provided in Appendix A.

Focus group interviews with therapists were conducted to encourage open discussion among peers, while individual interviews with leaders and non-therapist staff captured diverse organizational perspectives. All interviews were conducted by the first author via Microsoft Teams between January and June 2022. Two individual interviews were conducted by telephone—one because the participant was traveling without video access, and the other due to technical problems. Individual interviews lasted between 25 and 50 min, and focus groups lasted between 51 and 80 min. All interviews were audio-recorded and transcribed verbatim.

#### Observations in three clinics

The first author conducted one-day observations at each of the three clinics where the healthcare professionals worked and attended two 2-day network meetings involving all participant sites. The purpose of the observations was to identify organizational and contextual factors that influenced the implementation of guided iCBT and to complement the interview data. Across the clinics, the researcher observed clinical meetings, digital treatment sessions, and informal staff interactions to gain insight into everyday work and the practical organization of guided iCBT. The two network meetings provided organizational perspectives: one brought together senior mental health leaders to discuss digital care strategies, while the other brought team leaders together to share implementation challenges and reflect on leadership roles. During and after the observations, notes were taken manually and later analyzed alongside the interview data to contextualize the participants’ experiences.

#### Free-text responses from patients

Free-text responses were obtained from a routine post-treatment evaluation survey administered to patients receiving guided iCBT in four clinics. The survey included both structured and open-ended questions to assess patients’ experiences with the treatment, and the free-text responses constituted a secondary qualitative data source for the present study. The complete survey is provided in Appendix B.

### Participants

#### Healthcare professionals

Healthcare professionals were purposively selected from three clinics to capture experiences of implementing guided iCBT across different organizational settings and implementation practices. Participants were recruited based on their involvement in delivering, managing, or implementing guided iCBT. Therapists with varying levels of experience with guided iCBT and leaders from four organizational levels were included to ensure a diversity of clinical and organizational perspectives. The sampling strategy combined convenience, purposive, and snowball sampling. Initially, therapists and leaders were recruited through participating clinics. During data collection, additional non-therapist professionals with roles related to implementation and service delivery were identified and invited to participate. The final sample included therapists, leaders, and non-therapist professionals. An overview of participants’ roles is presented in Table 1.

**Table 1:** iCBT experience across participant groups.

Role	Number of participants	iCBT experience
Leaders	11	3
Therapists	15	11
Supporting Personnel	5	2
Total	31	16

#### Patients

Patients were recruited from four Norwegian clinics offering guided iCBT to capture a broad range of experiences from routine care. All patients who received treatment between September 2021 and June 2024 were invited to complete a post-treatment evaluation survey; 571 (50%) consented. The qualitative analyses were based on the 296 participants who provided at least one analyzable free-text response to the open-ended evaluation questions.

Participants included in the qualitative analyses were older, more likely to be self-referred, and had completed substantially more treatment modules than participants who did not provide open-ended responses. No significant differences were observed in gender, treatment program, or educational level (Table 2).

**Table 2:** Characteristics of all patients and comparisons between patients who did and did not respond to post-treatment open-ended evaluation questions (*N* = 571).

Characteristic	All (*N* = 571)	Patients with text response included in the analysis (*n* = 296)	Patients excluded due to missing open text response (*n* = 275)	*p*-value
Women, *n* (%)	360 (63)	188 (64)	172 (63)	0.811^#^
Age in years, Mean (SD)	33.0 (10.9)	34.5 (11.5)	31.3 (10.1)	<0.001*
Treatment program, *n* (%)				0.737^#^
Depression	275 (48)	143 (48)	132 (48)	
Panic disorder	138 (24)	75 (25)	63 (23)	
Social anxiety	158 (28)	78 (26)	80 (29)	
Patient reported referral type, *n* (%)				<0.001^#^
Self-referral	99 (17)	67 (23)	32 (12)	
GP referral	462 (81)	224 (76)	238 (87)	
Missing	10 (2)	5 (2)	5 (2)	
Education, *n* (%)				0.089^#^
Lower secondary school	41 (7)	21 (7)	20 (7)	
Upper secondary school	179 (31)	90 (30)	89 (32)	
Higher education (university/college)	181 (32)	112 (38)	69 (25)	
Other	27 (5)	12 (4)	15 (6)	
Missing	143 (25)	61 (21)	82 (30)	
Modules completed, mean ± SD*	4.4 ± 2.5	5.6 ± 2.2	3.1 ± 2.1	<0.001*

^a^The depression program consisted of 8 modules, whereas the panic disorder and social anxiety program comprised 9 modules. *Independent samples *t*-test between patients who did and did not respond to the post-treatment open-ended evaluation questions. ^#^*p-*value from Pearson’s chi-square test comparing the overall distribution between patients who did and did not respond to the post-treatment open-ended evaluation questions.

The qualitative material consisted of written responses to two open-ended questions from a post-treatment evaluation survey routinely administered after guided iCBT. The survey comprised 19 items assessing treatment outcomes, satisfaction, and experiences, using a combination of rating scales, yes/no questions, and open-ended responses (Appendix B).

To explore patients’ perceptions of expectations related to motivation and self-direction within guided iCBT, we focused on responses to two open-ended questions: (1) *“What do you think is the most positive outcome of the treatment? Provide a brief description*,” and (2) “*What do you think is the most negative outcome of the treatment? Provide a brief description*.”

These questions were selected to capture patients’ own descriptions of treatment experiences without imposing predefined categories. Because the survey was originally developed for routine service evaluation rather than for investigation of patient-role expectations, the patient material was treated as a secondary qualitative data source. The aim was not to examine patient-role negotiations directly but to explore how participants’ accounts of treatment experiences could provide insight into how expectations regarding autonomy, responsibility, effort, and support were responded to in practice.

Among the included participants, 292 responded to the positive outcome question and 216 to the negative outcome question. A total of 309 responses to the positive outcome question and 226 responses to the negative outcome question were collected.

Responses were screened for substantive content, and entries with no or minimal substantive content (e.g., “I do not know”) were excluded. This resulted in 296 responses to the positive item and 179 responses to the negative item being retained for analysis (Table 3).

**Table 3:** Characteristics of responses to post-treatment open-ended questions regarding positive and negative outcomes of the guided iCBT program (*N* = 296).

Characteristic	Reported positive outcomes^a^	Reported negative outcomes^b^
Patients providing free-text response, *n* (%)	292 (99)	216 (73)
Patients with ≥ 2 free-text responses to the same item^c^, *n* (%)	16 (5)	9 (4)
Total number of free-text responses (*n*)	309	226
Free-text responses analyzed^d^ (*n*)	296	179
Length of analyzed responses (in words)		
Mean (SD)	24.6 (23.0)	25.7 (26.7)
Median	18	18
Range	1–206	2–240

^a^Response to the question “What do you think is the most positive outcome of the treatment? Provide a brief description.” ^b^Response to the question “What do you think is the most negative outcome of the treatment? Provide a brief description.” ^c^Responded multiple times to the questionnaire. ^d^Free-text response with substantial content; remaining after removing text responses such as “I do not know,” “I do not have any negative/positive outcomes,” and similar. Comments with 1–2 words were included if they provided substantive input.

The unit of analysis was the individual retained free-text response. A small proportion of participants completed the evaluation questionnaire more than once, resulting in multiple responses from the same individual (Table 3). Repeated submissions could be identified through participant identifiers and reflected either participation in different treatment programs or repeated completion of the same program. Because the aim was to explore experiences associated with participation in guided iCBT, responses were retained as separate textual responses. The comments were generally brief, with a median length of 18 words for both questions, although some participants provided substantially longer reflections (Table 3).

### Analysis

Data from interviews and observations with healthcare professionals were analyzed using reflexive thematic analysis (Braun and Clarke, 2006, 2022), chosen for its flexibility and emphasis on researcher reflexivity. The analysis was primarily inductive, with themes developed from the data rather than guided by pre-existing theoretical frameworks. Following Braun and Clarke’s iterative six-stage process, with movement back and forth between stages, transcripts and field notes were read multiple times, and the first author documented initial reflections. Coding was conducted in two rounds, leading to the development of initial themes, which were subsequently reviewed and refined in collaboration with co-authors. This analytic process identified themes related to both the therapist’s role (discussed elsewhere; Standal et al., 2026) and the patient’s role, the latter of which is the focus of the present paper.

Free-text survey responses were analyzed using inductive qualitative content analysis (Graneheim and Lundman, 2004). Qualitative content analysis was considered appropriate because the patient material consisted of brief written comments rather than rich interview data, making a more descriptive analytic approach suitable. Responses were read repeatedly to achieve familiarity with the material and were coded inductively. Codes reflecting similar aspects of participants’ treatment experiences were grouped into categories, which were subsequently interpreted and abstracted into broader themes. Microsoft Excel and Microsoft Word were used to organize and iteratively refine codes and themes. Preliminary themes for the most positive outcomes included: *I’ve gained new skills*, *Getting better, Negative effects*, and *Others*. For the most negative outcomes, preliminary themes included: *It’s all too much, I did not do enough, Staying the same or getting worse, Low perceived suitability and confidence in the intervention, I needed more support*, and *Nothing.* All preliminary themes and their descriptions are presented in Appendix C.

Theme selection occurred prior to the application of Self-Determination Theory, which was used only during interpretation. Preliminary themes relating primarily to treatment outcomes (e.g., improvement, deterioration, or no change) were not retained because the analysis focused on participation, responsibility, autonomy, and support. Low perceived suitability and confidence in the intervention was also identified as a preliminary theme. Although important, these accounts were analytically heterogeneous and encompassed issues such as treatment fit, preferences, symptom-related challenges, and perceptions of the intervention. Rather than forming a coherent role-related theme, they informed interpretation of competence, treatment demands, and support needs.

The final themes selected for in-depth analysis were: *(1) I’ve gained new skills*, *(2) It’s all too much*, *(3) I could have done more*, and *(4) I needed more support*. These themes captured key dimensions of patients’ understanding of their roles, efforts, challenges, and support needs during treatment.

The resulting themes were subsequently interpreted in relation to patient-role expectations and self-determination theory.

**Figure 1:**
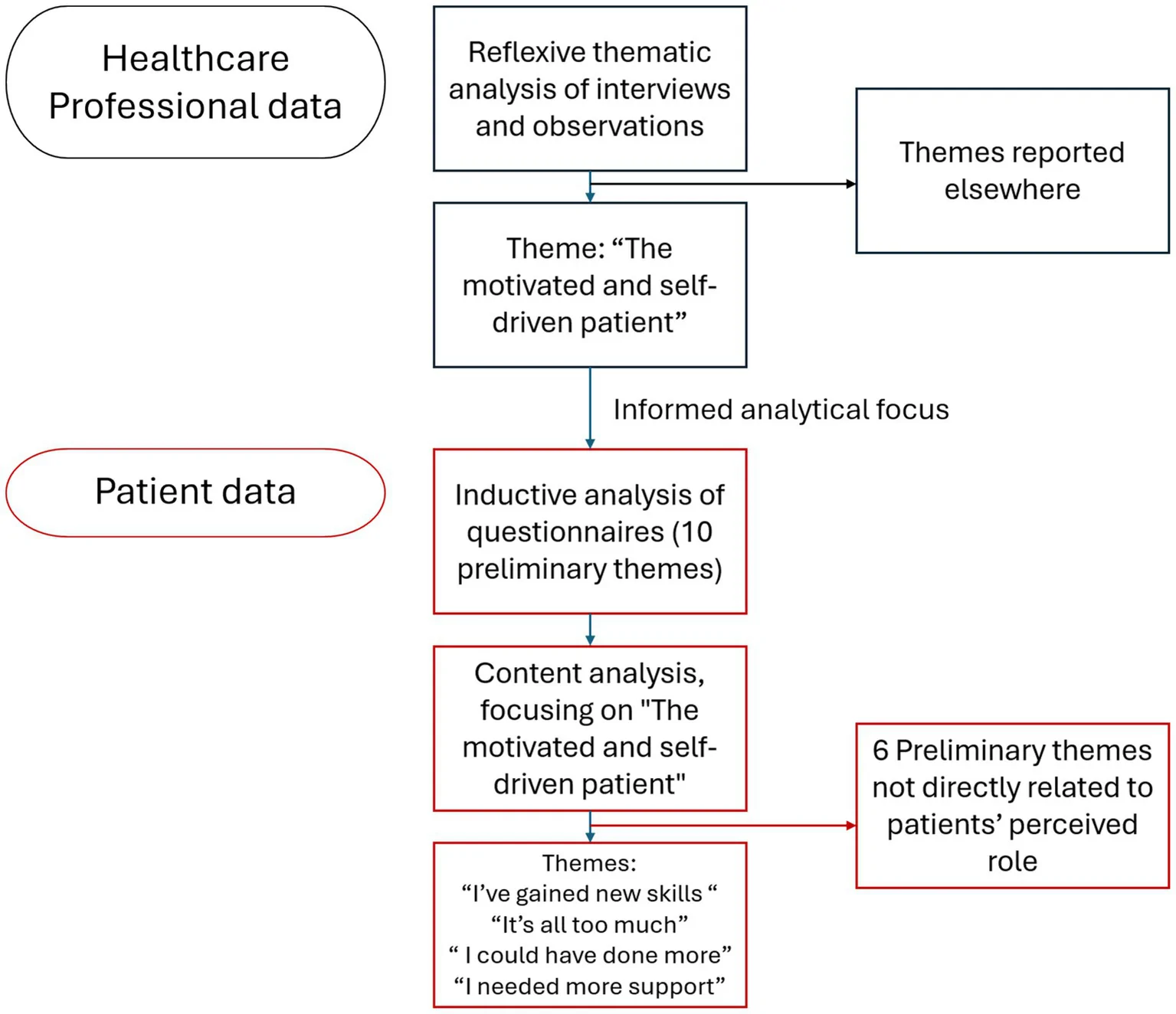
Overview of the analytic process across data sources. Insights from analyzing healthcare professionals’ perspectives informed the analytic focus on patient data, enabling a targeted exploration of how patients understood and enacted their role in guided iCBT.

Together, the two analyses provided complementary perspectives on the patient role in guided iCBT, combining insights into how it is conceptualized within the treatment context and how patients experience it. The analytic process is illustrated in Figure 1.

### Reflexivity and ethical considerations

The first author, a clinical psychologist with a longstanding interest in guided iCBT and prior involvement in introducing this treatment in Norway, recognized that her professional background and familiarity with several of the professional participants could influence the research process. To address this, she documented preconceptions before data collection and engaged in ongoing reflexive practice through written notes and regular discussions with co-authors during analysis.

All participants received written and oral information about the study and gave informed consent. The data were anonymized. Ethical approval for the healthcare professional data was assessed by the Regional Committees for Medical and Health Research Ethics (case number 396856), which concluded that no further approval was required. Approval for patient data was granted by the same committee (case number 229387).

## Results

The results are divided into two sections. The first explains how healthcare professionals conceptualized the patient’s role in guided iCBT. The second summarises participants’ descriptions of expectations, involvement, challenges, and perceived responsibilities.

The patient themes are based on free-text responses from 296 participants who provided analyzable responses to the post-treatment evaluation. Compared with patients who did not provide analyzable free-text responses, these participants were older, more often self-referred, and had completed substantially more treatment modules (Table 2). No significant differences were observed in gender, treatment program, or educational level. These findings suggest that the qualitative material primarily reflects the experiences of a relatively treatment-engaged subgroup of patients.

The themes identified across the healthcare professional and patient datasets are described in Table 4 and elaborated in the following sections.

**Table 4:** Themes related to the patient role in guided iCBT.

Source	Theme	Brief description
Healthcare professionals	The motivated and self-driven patient	Healthcare professionals described guided iCBT as placing expectations on patients to be motivated and self-directed, with responsibility for carrying out much of the treatment independently.
Patients	I’ve gained new skills	Participants described gaining new tools and a better understanding of their difficulties. Several participants reported feeling less alone by recognizing that their reactions were common and shared by others.
Patients	It’s all too much	The intervention was experienced as stressful and guilt-inducing, with demanding tasks that felt overwhelming and difficult to balance with everyday responsibilities.
Patients	I could have done more	Participants described disappointment in their own efforts, often accompanied by shame, low confidence, and a sense of failure.
Patients	I needed more support	Participants experienced insufficient therapist contact and an overly unstructured online format and expressed a need for more regular check-ins and clearer deadlines to support motivation.

### Professional conceptualizations of the patient role: “the motivated and self-driven patient”

Across the three clinics, healthcare professionals consistently described guided iCBT as a “suitable” option primarily for a specific patient group, those perceived as sufficiently motivated, self-driven, and able to handle the flexible treatment structure. The term “suitable” appeared repeatedly in interviews and observations, and was used as a criterion for assessing whether a patient was likely to benefit from the therapy:

*“…it is a good treatment for those it suits, as we usually say.”* (Leader 10).

Professionals emphasized that guided iCBT was most effective for patients who are both motivated and able to manage the treatment independently. Motivation was commonly described as central to success. Several clinicians emphasized that empowering patients and encouraging self-responsibility are essential principles in implementing guided iCBT. However, some acknowledged the challenges of expecting high levels of autonomy and self-management from individuals experiencing low energy, low motivation, or cognitive difficulties often associated with depression. While many professionals saw patients’ motivation and self-management capacity as given and necessary preconditions for therapy, some questioned whether these expectations are realistic for all patients. Observational data further illustrated that motivation often influenced referral decisions, sometimes overshadowing other considerations such as patient resources, digital literacy, or clinical complexity. During one iCBT team meeting, a therapist from another team was invited to discuss the possibility of referring one of their patients to the guided iCBT program. The iCBT team members were concerned about the patient’s ability to remain engaged, particularly during the behavioral activation phase. The referring therapist, however, argued that the patient was “*motivated enough*,” and allowed perceived motivation to override concerns about symptoms or other issues. Motivation thus became not simply one of many factors, but the primary criterion for offering guided iCBT. The interviews also reflect the view that a self-driven, motivated patient is essential for successful treatment results. This focus on patient responsibility is clearly articulated by one leader, who remarked that:

*“[…] that one makes the patient responsible, and the personal effort one requires from the patient is very important to succeed with the treatment.”* (Leader 11)

Across accounts, responsibility was commonly described as linked to patients’ motivation and their ability to take initiative and actively participate in therapy. Several clinicians further suggested that these expectations may be especially high in guided iCBT, where patients decide the pace and initiate therapeutic contact. Several informants noted that providing clear information about the structure and expectations of guided iCBT is crucial for both referrers and patients to evaluate whether the treatment aligns with the patient’s motivation and level of engagement. As one leader explained:

*“That is why it is important [….] with good marketing and that patients are well acquainted with what this is […]. Moreover, if patients then wish to try, and are in the target group, that is, they have the right diagnosis that the treatment is made for, […]. then it can be worth a try.”* (Leader 2)

Healthcare professionals across the clinics identified three criteria for determining suitability: (1) having one of the specified diagnoses, (2) not having severe or complex conditions, and (3) showing enough motivation and self-direction to complete the treatment with minimal therapeutic support. Among these, motivation and self-direction were consistently seen as the most important criteria. At two clinics, self-referral pathways were described as particularly effective for identifying patients who actively chose guided iCBT and were therefore perceived by participants as more motivated and better equipped to engage with and complete the treatment. These patients were assumed to have a higher level of intrinsic motivation, and clinicians viewed them as more likely to complete the modules and benefit from asynchronous therapy:

*“…the patient who [self-referred], I find that they can go through with and follow up the program to a greater extent.”* (iCBT therapist 23)

Overall, healthcare professionals’ accounts suggest that the “motivated and self-driven” patient functions as an implicit ideal within guided iCBT. Expectations regarding suitability, autonomy, responsibility, and motivation were actively negotiated through referral processes and treatment decisions, shaping how professionals understood who was likely to engage with and benefit from treatment. The patient role therefore emerged not as a fixed characteristic but as one constructed through organizational and clinical assumptions about the demands of guided iCBT. The following section examines how patients themselves described and experienced their role within this treatment model.

### Patient perspectives: four themes complicating the ideal of the self-driven patient

Analysis of patients’ free-text responses to the survey identified four themes that both expand and challenge the professional perspective. Together, these themes show how patients interpret their efforts, struggles, and support needs throughout treatment.

As noted above, the patient themes primarily reflect the experiences of a relatively engaged subgroup of patients.

#### Theme 1: I’ve gained new skills

This theme reflected a recurring pattern in patient responses. When asked about the most positive effects of the treatment, several responses emphasized that the guided iCBT program had provided them with new skills and a deeper understanding of their psychological difficulties. Patients also emphasized the importance of acquiring and applying skills to manage their difficulties independently.

Patients reported gaining substantial insight into their symptoms and feeling more capable of managing their psychological difficulties independently. Several patients described that the treatment program helped them better understand their experiences and provided them with practical strategies they had not previously used or felt they could use. As one patient described:

*“I have learned more about anxiety and why it occurs, as well as what happens in the body when anxiety arises. I have also learned that these reactions are completely normal and not dangerous. I now understand more about how thoughts work, how they can influence the body and brain, and how everything is connected. This has shown me that simply changing one’s thinking can make a significant difference. As a result, I find it easier today to accept my anxiety than I did before.”* (Patient 3,231).

This quote about symptoms and new insights reflected what patients commonly described as the central value of the treatment program: gaining knowledge and specific skills that foster a more accepting approach toward their psychological difficulties. Patients emphasized that they learned practical tools they could continue to use independently after the treatment sessions, enabling them to handle challenges, as illustrated by another participant:

*“The most positive aspect is that I have gained useful tools that enable me to do what I can on my own.”* (Patient 3,048).

Patients reported that these tools not only provided immediate support but also strengthened their capacity to actively engage with their difficulties over time. Several described an enhanced sense of autonomy, noting that they no longer felt dependent on external support to manage their mental health. They described having gained more control, as one patient summarised:

*“The most positive outcome of the treatment is that I feel I have greater control over how my days unfold. I have gained useful tools through the activity plan and in managing negative automatic thoughts, and I intend to continue using these even after the treatment has ended.”* (Patient 3791)

Patients reported that guided iCBT offered tools and strategies they could continue to use on their own and expressed confidence in applying them after treatment. They also described that the insights and strategies acquired during the sessions would support long-term coping and progress beyond the treatment period. Overall, patients regarded the skills acquired through guided iCBT as empowering. However, the next theme reveals that this sense of autonomy can also feel overwhelming and at times difficult to manage.

#### Theme 2: it’s all too much

While the previous theme highlighted how patients valued gaining skills and a sense of control, this theme presents a contrasting perspective. When asked about the most negative aspects of the treatment, some patients described it as demanding and at times overwhelming. These demands often caused stress and guilt, particularly when patients struggled to balance treatment requirements with their daily responsibilities. Responses described the program as “a lot of work,” characterizing the intervention as stressful and guilt-inducing, noting that exercises and tasks could become overwhelming and difficult to integrate into daily obligations. The following quote illustrates this workload:

*“You must spend a lot of time on it. If you have a child, a job, and so on, it is at times impossible to keep up with the treatment.”* (Patient 2604)

The patient articulated a view commonly shared: the treatment is highly time-consuming, making it difficult to follow the program as intended alongside the demands of everyday life. Several patient responses indicated that the treatment was causing emotional strain. They described the treatment as emotionally demanding, particularly the exposure-based exercises that required them to confront difficult thoughts, feelings, or situations. One participant remarked:

*“It has sometimes been tough to enter exposure situations on bad days when I haven’t felt well.”* (Patient 3523)

This statement reflected how patients experienced the treatment as emotionally demanding, particularly during exposure exercises. Several patients described the treatment as exhausting, both mentally and physically. At the same time, the patients also showed a dual awareness of the burdens and benefits of therapeutic work, suggesting that while the process may be demanding, it is also perceived as meaningful.

Several patients mentioned that they would have wanted more time and flexibility to pause treatment during holidays, sick leave, or other life events. Patients reported that they had too little time to complete the treatment, which added to their stress and sense of pressure. One patient described how they felt time pressure:

*“It became too time-consuming at times, especially when I was unwell, and I felt guilty for not keeping the promises I made in the activity plan.”* (Patient 1430)

This quote illustrated how rigid treatment structures can unintentionally contribute to feelings of inadequacy and guilt, as they fail to accommodate the reduced and fluctuating energy levels associated with patients’ psychological conditions. As a result, the guilt they experienced became an additional burden produced by the structure of the intervention.

Taken together, this theme showed how the treatment’s demanding structure can feel overwhelming and add feelings of guilt for patients. The following theme further demonstrates how patients may internalize these structural challenges, leading them to believe they should have done more themselves.

#### Theme 3: I could have done more

The previous theme’s focus on guilt and inadequacy provides the context for this next theme. It explores how patients may view their difficulties as personal shortcomings, believing they should have done more. When asked about the most negative outcome of the treatment, several participants described difficulties in initiating or maintaining engagement with it. They articulated that these issues were due to personality traits such as procrastination or low self-discipline, rather than to the structure of the program, as highlighted by this patient:

*“It gave me a bit too much freedom, which, with my messy brain, meant I couldn’t complete everything as I should have, and I’m a bit annoyed with myself for that.”* (Patient 2947)

This account suggested that the program was intentionally designed to promote autonomy. However, the participant blamed their failure to follow it on personal shortcomings rather than on its design, internalizing responsibility and criticizing themselves for not meeting all the requirements. This self-blame was also associated with feelings of guilt and internal conflict, as shown by another participant’s experience:

*“I’ve felt some guilt because I procrastinate so much and haven’t done as much of the treatment as I wanted to. So, it’s always been a task hanging over me, without me doing much about it.”* (Patient 3186)

Here, the patient attributed their struggles not to the program’s format or workload but to perceived personal deficiencies, such as procrastination and a lack of discipline. This attributional pattern shifted responsibility inward: rather than recognizing structural factors that might hinder participation, the patient assumed sole responsibility for managing and resolving these difficulties. In this way, the burden of overcoming obstacles to engagement was internalized, reinforcing a sense of personal inadequacy.

Overall, this theme showed how patients experienced inadequate effort, accompanied by shame and self-criticism. This self-blame suggested that autonomy is interpreted as an individual obligation, where unmet expectations lead to guilt. It also pointed to a need, explored in the following theme, for increased therapist contact to provide guidance and reassurance.

#### Theme 4: I needed more support

In patient accounts, engagement issues are sometimes framed as personal shortcomings, while structural aspects are also highlighted in descriptions of what affects motivation. This theme of “needing more support” captures a recurring concern in the material: insufficient therapist contact and the need for more regular check-ins and clearly defined deadlines. These elements are described as essential for maintaining the structure that sustains motivation and enables progress. Several participants expressed that they wanted more face-to-face interaction with a therapist, describing that the digital format made them feel lonely or lacking support. One participant stated:

*“The most negative thing about the treatment is that everything is online. I wish there had been one or two meetings where we could meet the therapist to talk about progress or setbacks.”* (Patient 2999)

Several participants expressed a similar desire for more in-person meetings during treatment, highlighting the limitations of purely digital interventions, especially in providing emotional support and strengthening the therapeutic alliance. While several participants appreciated the convenience of digital sessions, they also noted that these lacked the emotional connection and support usually present in traditional face-to-face therapy. They described something crucial as missing: the sense of being fully understood and supported by another person. In addition to emotional support, participants emphasized the importance of personal contact with a therapist to tailor treatment. When asked about the most negative aspect of the treatment, one participant responded:

*“Not being able to meet a psychologist face-to-face — I think it would have helped to speak with someone who has the expertise to understand me and my challenges beyond this more “template-based” approach.”* (Patient 3161)

According to this patient, the program’s pre-written and standardized structure made the treatment feel too generic to address her/his unique issues effectively. Similar accounts highlighted that seeing a therapist would have enabled them to articulate their challenges more openly and receive a deeper level of understanding and responsiveness that the generic content could not provide. Several suggested that more frequent contact with therapists could compensate for this lack of personalization by providing tailored guidance and recognition of their specific circumstances.

Taken together, these accounts showed that both individual challenges and structural features, particularly limited therapist contact and perceived low personalization, played a role in shaping patient engagement.

## Discussion

This study shows how healthcare professionals and patients perceive and interpret participation in guided iCBT in relation to expectations about the patient role. The findings highlight how expectations of autonomy and responsibility are articulated, interpreted, and at times perceived as challenging. Healthcare professionals consistently described a patient role involving motivation and self-direction, while patients’ perspectives both support and challenge this view. Together, these findings demonstrate that responsibility for managing treatment is understood as a balance between individual effort and treatment structure.

Importantly, processes that may be interpreted as negotiation of the patient role appeared differently across the two datasets. Among healthcare professionals, negotiation was visible in discussions regarding patient suitability, responsibility, referral decisions, and expectations of autonomy. Among patients, participants’ accounts of self-directed participation provided insight into how they understood, responded to, and positioned themselves in relation to associated expectations. From the perspective adopted in this study, these responses may be interpreted as expressions of negotiation.

The theme *“I’ve gained new skills”* illustrated how patients perceived themselves as acquiring valuable knowledge and competencies through the therapeutic process. By emphasizing these new skills as positive outcomes, they implicitly viewed themselves as active participants responsible for their own progress. However, this sense of responsibility could also create pressure. In the theme *“It’s all too much,”* patients described feeling overwhelmed by the task of managing treatment on their own. The theme “*I could have done more”* showed self-criticism and disappointment, indicating that patients blamed themselves for the progress or the lack of it, and saw this as their responsibility. Meanwhile, in the theme *“I needed more support,”* several patients expressed that they would have benefited from more personalized guidance to help them stay motivated and engaged throughout treatment.

The patient role was described in ways that suggest it is continually worked out through the interaction between institutional expectations and patients’ own experiences of participating in treatment. In this sense, the findings align with social constructionist understandings of roles as shaped and negotiated in practice (Berger and Luckmann, 1966; Wenger, 1999).

The findings should also be considered in light of the composition of the patient sample. Patients who provided analyzable free-text responses had completed substantially more treatment modules than those who did not, suggesting that the patient material primarily reflects the experiences of individuals who remained sufficiently engaged to participate in both treatment activities and the post-treatment evaluation. Notably, themes relating to feeling overwhelmed, experiencing self-criticism, and wanting more support emerged even within this relatively engaged subgroup. It is therefore possible that the challenges associated with treatment participation are not overstated in the present material and may, in fact, be underrepresented. Patients who disengaged earlier, completed fewer modules, or did not respond to open-ended questions may have experienced these demands differently or more strongly.

### Interpreting the findings through self-determination theory

To interpret these findings, we draw on Self-Determination Theory (SDT) (Deci and Ryan, 1985; Ryan and Deci, 2020). SDT emphasizes motivation and self-direction, assuming that patients are more likely to participate in treatment when they experience a sense of autonomy. This corresponds to the concept of autonomous motivation, whereby patients engage in treatment because they personally value and endorse it, rather than because of external pressure or obligation. According to SDT, autonomous motivation is sustained when three basic psychological needs are supported: autonomy, competence, and relatedness (Ryan and Deci, 2006). This framework provides a useful lens for understanding how these needs can be supported or undermined by guided iCBT.

#### The dual features of autonomy

Both healthcare professionals and patients viewed guided iCBT as a format that assumes and requires a high degree of autonomy. Many patients valued the flexibility and independence to organize their treatment, consistent with previous research highlighting the importance of autonomy in digital interventions (Etzelmueller et al., 2018; Lilja et al., 2021). The option to access treatment materials flexibly, such as downloading content for later use, is valued by patients (Hadjistavropoulos et al., 2018); this flexibility may be understood as supporting autonomy. Autonomy has been identified as an outcome of guided iCBT (Bendelin et al., 2011), and a recent study indicates that therapist support can help strengthen patients’ sense of autonomy during treatment (Seittu et al., 2025).

At the same time, our findings suggest that features intended to support autonomy may not always be perceived as supportive. When autonomy is assumed instead of actively encouraged, it may be experienced as demanding or burdensome. This concern echoes findings from qualitative studies in which patients described flexibility as both beneficial and burdensome, particularly when self-management demands exceeded available support (Hadjistavropoulos et al., 2018; Gericke et al., 2021). This was particularly evident in the theme “*I could have done more*,” where patients frequently interpreted difficulties with engagement as personal failure, expressing guilt, self-criticism, and disappointment in themselves rather than attributing these challenges to the demands of the treatment format. This suggests that expectations of self-direction may, for some patients, become internalized as pressure rather than experienced as autonomy support. These findings highlight the importance of differentiating between autonomy as an inherent part of the treatment’s structure and autonomy as a psychologically supported experience.

#### The complexity of building competence

Patients frequently described gaining new skills and understanding, which fostered a sense of competence. Their emphasis on applying therapeutic strategies independently suggests that expectations about individual responsibility may be adopted during treatment. This finding aligns with studies showing that guided iCBT can strengthen perceived ability to manage symptoms (Axelsson et al., 2020; Gericke et al., 2021; Terides et al., 2018). At the same time, research by Berg et al. (2019) suggests that increases in knowledge constitute a distinct construct that does not necessarily translate into symptom improvement. Our study adds nuance to these findings by showing that many patients described gaining skills and understanding, whereas others found it difficult to handle treatment demands. In these situations, competence appeared less supported, and patients reported frustration, self-blame, and disengagement. Preliminary analyses also identified accounts of low perceived suitability for the intervention. Participants described the treatment as overly generic, insufficiently tailored to their particular difficulties, difficult to follow because of concentration problems, and at times challenging to navigate due to the amount and structure of written material. Although these experiences were not developed into a separate theme, they further suggest that patients differed in the extent to which they experienced themselves as capable of meeting the demands embedded within guided iCBT.

#### Relatedness: the challenges with limited support

Patients’ descriptions of wanting more personalized and responsive therapist contact may be understood in relation to the psychological need for relatedness, which, in SDT, is considered important for sustaining autonomous motivation and well-being. While some studies have found that the quality of the therapeutic relationship is not a strong predictor of treatment outcome in guided iCBT (Hadjistavropoulos et al., 2017; Knaevelsrud and Maercker, 2006), others highlight the importance of the early working alliance (Axelsson and Hedman-Lagerlöf, 2025). Zalaznik et al. (2025) distinguish between alliance with the program and alliance with the therapist in guided iCBT, suggesting that patients form relationships with both components. Both types of alliance have been linked to adherence and dropout, whereas therapist alliance is more strongly associated with symptom improvement (Zalaznik et al., 2025). Similarly, Seittu et al. (2025) emphasize the therapist’s role in strengthening the therapeutic alliance and in supporting patients’ sense of autonomy and emotional regulation, highlighting the continued importance of relational processes in digital interventions. Patients in our study frequently expressed a desire for more personalized and responsive interactions, highlighting the tension between relational needs and the efficiency-driven, standardized design of digital interventions. This finding is consistent with Sayar et al. (2023), who reported that a subset of patients in guided internet interventions described unmet expectations regarding therapist availability, emotional support, and individualized guidance. Previous studies have similarly shown that patients value individualized feedback, responsiveness, and opportunities for clarification within digital treatment programs (Hadjistavropoulos et al., 2018; Perera-Delcourt and Sharkey, 2019).

#### When psychological needs are unmet

From an SDT perspective, difficulties sustaining engagement in guided iCBT are closely related to the extent to which patients’ basic psychological needs for autonomy, competence, and relatedness are supported within the treatment context (Ryan and Deci, 2017; Ryan and Deci, 2020). When these needs were insufficiently supported, patients in our study described experiencing strain, self-doubt, and reduced engagement.

Descriptions of inadequacy and emotional strain often accompany difficulties in meeting expectations associated with the self-directed patient role. In SDT terms, this pattern may reflect a shift from more autonomous to more controlled forms of motivation (Ryan and Deci, 2020), in which engagement is driven less by volition and more by internal pressures such as self-criticism and obligation.

Importantly, these experiences were rarely attributed solely to the treatment structure itself. Instead, several patients described their difficulties as personal shortcomings, such as insufficient discipline or effort, which may suggest that professional expectations of motivation and self-direction had been adopted and reproduced in their own accounts. From an SDT perspective, this resembles *introjection*, in which external expectations are internalized without being fully integrated, often resulting in psychological costs such as anxiety, self-blame, and diminished well-being (Ryan and Deci, 2020).

This study broadens prior research on unmet needs in digital therapy (e.g., Sayar et al., 2023) by incorporating both healthcare professionals’ perspectives and patients’ experiences. The findings suggest that these challenges may be understood not solely in relation to individual factors or therapist support, but also in relation to role expectations, treatment structures, and relational conditions.

Guided iCBT may be understood as organizing treatment in ways that place substantial responsibility on patients to manage treatment activities while therapists function primarily as guides. Responsibility for both symptom management and treatment engagement may therefore be experienced as resting largely with the patient. Patients’ accounts of “missing” therapist contact, together with their tendency to attribute difficulties to themselves, may be understood as reflecting tensions between expectations of self-direction and desires for more relational forms of support. This interpretation resonates with findings from Sayar et al. (2023), but extends this work by suggesting that such experiences are related not only to therapist contact itself, but also to how responsibility and autonomy are organized within guided iCBT.

More broadly, these findings can be situated within wider shifts in digital health, where patients are increasingly expected to act as what Lupton (2017, p. 43) terms the “responsible healthy citizen,” taking on greater responsibility for managing their own care. While guided iCBT aligns with these ideals by encouraging active and self-directed participation, the findings suggest that autonomy may at times be treated as a presupposed capacity rather than something actively supported, potentially contributing to negative treatment experiences or disengagement.

Taken together, these findings suggest that unmet psychological needs in guided iCBT are not only individual experiences but also shaped by how the patient role is constructed within the treatment. In this way, unmet needs can be understood not simply as a lack of support, but as an outcome of how autonomy, competence, relatedness, and responsibility are distributed within the treatment.

### Are these findings specific to guided iCBT?

An important question is whether the tensions identified in this study are specific to guided iCBT or reflect broader features of structured psychological treatments. Expectations of active participation, homework completion, behavioral change, and personal responsibility are central to CBT more generally and have also been described in face-to-face settings. Research on standardized group CBT has similarly shown that patients differ in their needs and that standardized treatment formats can make personalization difficult (Gryesten et al., 2024). Together, these findings suggest that some of the tensions identified in the present study may reflect broader features of structured and manualized psychological treatments rather than internet delivery alone.

At the same time, guided iCBT organizes these expectations in distinctive ways. Treatment relies heavily on independent completion of structured modules, asynchronous written communication, regular completion of symptom assessments, and patient-initiated help-seeking, while opportunities for real-time interaction and clarification are more limited than in face-to-face, blended, or videoconference-based therapy (Andersson et al., 2019; Etzelmueller et al., 2018; Thew, 2020). Patients’ descriptions of insufficient support, limited personalization, and difficulties managing treatment demands are consistent with previous findings that a subset of patients in guided internet interventions report unmet needs regarding therapist contact, responsiveness, and emotional support (Sayar et al., 2023). Patients’ experiences in the present study may therefore reflect both CBT-related expectations and the specific way responsibility and support are organized within digitally delivered treatment.

### Understanding engagement across multiple levels

The findings are consistent with an understanding of motivation as shaped by interactions between patients and their treatment context rather than solely by stable individual characteristics. Accordingly, a self-determination theory perspective highlights how treatment structures, therapist support, and organizational expectations may influence patients’ opportunities to sustain motivation and participation (Ryan and Deci, 2017; Ryan and Deci, 2020). Interestingly, several patients in the present study explained difficulties in terms of personal shortcomings such as procrastination, insufficient effort, or lack of discipline. In contrast, our interpretation suggests that these experiences should also be understood in relation to the demands embedded within the treatment format and the support available for meeting them. From this perspective, motivation may be understood as co-produced through the interaction between individual capacities and the treatment context rather than as a fixed patient trait.

Consistent with this perspective, responsibility for participation in guided iCBT cannot be understood solely at the level of the individual patient. Difficulties with engagement appeared to be associated with factors operating at multiple levels, including patient-level factors such as symptoms, concentration difficulties, self-regulation, and competing life demands; treatment-level factors such as workload, pace, homework, and module requirements; therapist-level factors such as responsiveness, personalization, and relational support; and organizational factors shaping referral decisions and treatment access. In addition, aspects of the digital treatment format, including asynchronous communication and limited opportunities for immediate clarification, may influence how demands and support are experienced. This perspective highlights how challenges associated with participation emerge across interacting patient, treatment, therapist, digital, and organizational contexts.

### Strengths and limitations

A key strength of this study is its use of qualitative analysis across multiple data sources, including therapists’ accounts of routine clinical practice, within-clinic observations, and patient free-text responses. Integrating these perspectives enabled a more comprehensive understanding of participation in guided iCBT. In addition, self-determination theory provided a coherent analytical lens for examining how autonomy, competence, and relatedness are supported or constrained within this treatment format.

However, several limitations should be considered. First, the first author was responsible for data collection and the initial stages of analysis and had prior experience in one of the clinics. While this facilitated access and insight into the setting, it may also have influenced data generation and interpretation despite ongoing reflexive efforts. Second, the study was conducted in services that had already implemented guided iCBT, and all participating patients had agreed to engage in this form of treatment. This may have resulted in a sample more positively disposed toward the intervention, potentially limiting insight into challenges related to uptake and continued participation. Additionally, patients who provided free-text responses differed from non-responders in that they were older, more often self-referred, and had completed more treatment modules. This suggests that the qualitative sample may overrepresent patients who were more engaged with the treatment. Consequently, the findings may not fully capture the perspectives of patients who disengaged or discontinued treatment, and their experiences may therefore be underrepresented. At the same time, the fact that themes related to burden, self-criticism, and unmet support needs emerged among participants who generally appeared more engaged with treatment suggests that these challenges may also be relevant beyond the less-engaged patient groups underrepresented in the present material. Thirdly, the two datasets were collected over partly different time periods. Interviews and observations with healthcare professionals were conducted in 2022, whereas patient survey responses were collected between 2021 and 2024. Although the services operated within the same implementation context, changes in clinical practice, experience with guided iCBT, or organizational procedures over time may have influenced the perspectives reported in the two datasets.

Finally, although patient perspectives were captured through free-text responses, these were originally collected as part of a routine treatment evaluation rather than a study of patient-role expectations. The comments were generally brief (median length 18 words), limiting opportunities to explore participants’ experiences, interpretations, and contextual meanings in the depth typically afforded by qualitative interviews. Consequently, the comments did not allow the same depth of exploration as qualitative interviews. Also, they provided only retrospective accounts of how participants interpreted and responded to expectations regarding participation, autonomy, responsibility, and support.

### Implications

Our findings have implications for both treatment allocation and service delivery. Assumptions about patient readiness and self-direction did not always align with patients’ experiences, suggesting that greater attention to how structure, guidance, and relational support are provided may be important for accommodating diverse needs within guided iCBT. In the professional data, perceived patient motivation was often described as a central criterion for determining suitability for guided iCBT and, in some instances, appeared to outweigh other clinical considerations. This suggests that suitability may be influenced not only by diagnosis, symptoms, and patient preference, but also by clinicians’ expectations regarding a patient’s anticipated ability to engage with the treatment format. While such assessments may reflect legitimate concerns about treatment fit, they raise important practical and ethical questions regarding access to digital interventions. Greater transparency regarding the role of motivation in referral decisions, together with consideration of patient preferences, support needs, and readiness for self-directed treatment, may help ensure a broader assessment of suitability.

The findings also highlight the importance of designing services that support patients’ needs throughout treatment. Clear communication regarding treatment structure, workload, duration, and therapist availability may help patients make informed decisions about participation. Greater flexibility in pacing, opportunities to pause treatment during periods of increased life stress, and proactive therapist check-ins may support patients who find self-directed participation difficult to sustain. Some patients may also benefit from higher levels of guidance or alternative treatment formats when the demands of guided iCBT exceed their needs or capacities.

From a self-determination theory perspective, such adaptations may support autonomy through flexibility and choice, competence through clear expectations and manageable demands, and relatedness through responsive therapist support. Rather than treating motivation and self-direction as stable patient characteristics, services may benefit from considering how treatment structures and support arrangements shape opportunities for sustained engagement. Future studies designed specifically to investigate experiences of internet delivery may provide greater insight into issues such as usability, digital literacy, technical barriers, and platform-related engagement.

## Conclusion

This study shows that both health professionals and patients view guided iCBT as requiring a motivated, self-directed patient role. While autonomy and personal responsibility emerge as central expectations, patients’ accounts reveal differing experiences of these expectations, highlighting an inherent tension between autonomy and the need for structure, guidance, and relational support. From an SDT perspective, these findings suggest that autonomy within guided iCBT is not inherently experienced as supportive. However, its motivational value depends on whether it is accompanied by adequate support for competence and relatedness. When autonomy is presupposed rather than actively supported, it may be experienced as pressure, potentially contributing to self-criticism and disengagement.

Taken together, the study shows that guided iCBT not only depends on patient motivation but also shapes how motivation is experienced through the expectations and responsibilities associated with the patient role. Some patients who struggled to meet expectations of self-direction described these difficulties as personal failure rather than attributing them to features of the treatment structure, often expressing self-criticism, guilt, and disappointment in themselves. Interpreted through SDT, this pattern may reflect introjection, whereby expectations of autonomy and responsibility are internalized without being fully integrated, potentially resulting in psychological costs. These findings suggest that the challenge in guided iCBT is not only to support patient motivation, but also to ensure that difficulties with participation are not experienced as individual failure when they may partly reflect the demands and expectations embedded within the treatment context.

## Data Availability

The datasets presented in this article are not readily available because of confidentiality considerations but are available from the corresponding author upon reasonable request. Requests to access the datasets should be directed to beatestandal@hotmail.com.
