# Supplementary material for: The self-directed patient in guided internet-delivered CBT: a qualitative study of patient role expectations
Source: Front Psychol. 2026 Aug 19;17:1924186. doi: 10.3389/fpsyg.2026.1924186 (PMC13534039; doi:10.3389/fpsyg.2026.1924186)
Supplement: Supplementary file 1 [file Supplementary_File_1.docx]

# Appendices

## Appendix A: Interview guide

1. Can you tell me about your experiences with guided iCBT?

a. Can you exemplify how it is used in your organization?

2. What are your thoughts about providing guided iCBT?

a. Is it a tool that can be used? How and for whom?

3. If we take for granted that guided iCBT should be used, what do you think it takes for this tool to be used and be recognized and considered equal to other treatment methods?

a. What does it take in your daily work?

b. What does it take in (name of workplace)?

c. What does it take in (name of local health trust)?

d. What does it take in (name of regional health trust)?

e. What does it take on a national level?

4. Is there anything we have not talked about that you think is important regarding the introduction of guided iCBT?

5. Please mention three things that you think could make guided iCBT more widely used as a treatment method.

## Appendix B: Patient survey

**Evaluation**

People who have applied for treatment always form opinions about the healthcare service they receive. Below are some questions about the healthcare service you have received; please share your opinions and assessments. For each question, please **check the response alternative that best fits you.**

**General about the treatment**

1. All things considered, how much do you think you have changed in a positive direction?

 None

 A little

 Quite a lot

 Very much

2. What do you think is the most positive outcome of the treatment? Provide a brief description:

_______________________________________________________________

3. Have others expressed that you have changed positively?

 Yes No

4. Are you satisfied with these changes, or would you have liked to change even more?

 Very satisfied, have reached all goals

 Mostly satisfied, have reached most goals

 Dissatisfied, would like to have reached more goals

 Very dissatisfied, would like to have reached more goals

5. Do you think the treatment has had any negative effects on you and your life?

 Extensive negative effects

 Some negative effects

 No significant negative effects

 Only positive effects

6. If you have experienced negative effects of the treatment, describe them here: _________________________________________________________________________

7. Have others expressed that you have changed negatively?

 No Yes

8. What do you think is the most negative outcome of the treatment? Provide a brief description: _______________________________________________________________

9. Have you recently experienced difficulties that affected the treatment negatively?

No

Yes

 Relationship breakup

 Financial difficulties

 Own or others’ illness

 Problems at work/studies

 Religious crisis

 Other (What?) ____________________

10. How demanding did you find the treatment?

 Very demanding

 Quite demanding

 Moderately demanding

 Not demanding

11. Considering the results of the treatment, was it worth the effort you put in?

 Definitely worth the effort

 Not quite worth the effort

 Partly worth the effort

 Not worth the effort

12. Would you have had the same benefit from the treatment without guidance from a therapist?

 I would have done better without support

 I would have done just as well without support

 I would have done somewhat worse without support

 I would have done much worse without support

13. When you had problems or questions, how often did you ask your therapist?

 Never

 Rarely

 Sometimes

 Often

 Always

14. Was it easy or difficult to understand what the therapist wrote?

 Very difficult

 Somewhat difficult

 Reasonably easy

 Very easy

15. What did you miss in the contact with the therapist?

_______________________________________________________

16. If a friend had similar problems as you, would you recommend eMeistring?

 No, absolutely not

 No, I don’t think so

 Yes, I think so

 Yes, definitely

17. All in all, how satisfied or dissatisfied are you with the treatment in eMeistring?

- Quite dissatisfied
- Neutral or slightly dissatisfied

 Quite satisfied

 Very satisfied

18. Which digital device have you used in the treatment? Check all that apply.

 Desktop PC

 Laptop

 Tablet

 Mobile

19. Suggestions for improvement and other comments: _______________________________________________________

## Appendix C: Preliminary themes and descriptions

| Most positive outcome | | Most negative outcome | |
| --- | --- | --- | --- |
| **Preliminary theme** | **Description** | **Preliminary theme** | **Description** |
| I’ve gained new skills | Users highlighted the intervention as a useful tool that supported reflection, strengthened learning and understanding, and helped them feel less alone through a sense of normalization. They valued having continued access to the tool afterward, noting that it boosted their confidence and increased their motivation to keep working on their challenges. | It’s all too much | Users described the intervention as demanding and emotionally taxing, often leaving them stressed and burdened with a sense of guilt. The workload felt overwhelming, with long texts, difficult writing tasks, and activities that required substantial time and effort. Overall, many found the process exhausting and hard to manage alongside everyday responsibilities. |
| Getting better | Because of the treatment, participants experienced fewer symptoms and improved daily functioning; they especially highlighted acceptance as an important part of this. | I didn’t do enough | Users expressed disappointment in their own efforts, often accompanied by shame and low self‑confidence. Many described a sense of failure, reflecting a belief that they had not met their own expectations or achieved what they had hoped for. |
| Negative effects | When asked about the positive effects, some people instead reported negative experiences. They said the treatment did not suit them, that they stopped the program, noticed little improvement, or felt they had no positive effects at all. | Staying the same or getting worse | While some experienced improved symptoms or functioning, others did not notice any change or got worse during treatment. |
| Other | Other positive effects reported by patients include feeling a good connection with their therapist, appreciating their own effort, and experiencing a sense of being seen and heard. Some valued receiving help quickly, liked that the treatment was online, and felt it provided a solid and acceptable starting point for further treatment. | Low perceived suitability and confidence in the intervention | Some users reported low perceived suitability and limited confidence in the intervention, describing it as technically cumbersome, containing unnecessary information, too short, and generally a poor fit for their needs. They also felt the content was overly generic, and some expressed little belief in the treatment's potential to help them. |
|  |  | I needed more support | Some users expressed that they missed more regular contact and support from their therapist. Some also rejected the online format. They felt the structure was too open and unbound, with few scheduled conversations. Several said they would have preferred pre‑booked check‑ins. Some also wanted clearer deadlines, as the current level of freedom made it harder to stay motivated and feel adequately supported. |
|  |  | Nothing | About one‑quarter of participants (54 out of 222) reported either being unsure or experiencing no negative outcomes when explicitly asked about this. |
